# Near-infrared OCT imaging for the assessment of anisocoria

**DOI:** 10.1038/s41433-026-04536-8

**Published:** 2026-05-27

**Authors:** Aliza Geller, Ora Paltiel, Joshua M. Kruger

**Affiliations:** 1 Department of Ophthalmology, Hadassah-Hebrew University Medical Center, Jerusalem, Israel; 2 Braun School of Public Health and Community Medicine, Faculty of Medicine, Hadassah-Hebrew University of Jerusalem, Jerusalem, Israel

**Keywords:** Diagnosis, Pupil disorders

## Abstract

**Background:**

Assessment of anisocoria in clinical settings can be very challenging, particularly for young children and patients with dark irises. In some instances, a smartphone camera is used to capture an image of the pupils to aid in accurate assessment. This study aims to assess and compare the inter and intra-rater reliability of assessment of anisocoria using near-infrared optical coherence tomography (NI-OCT) and smartphone photographs.

**Methods:**

In this prospective cross-sectional study, binocular photographs of thirty participants, aged eight months to sixty-eight years, were taken at four time points using smartphone and NI-OCT cameras after the application of Tropicamide 0.5% to create varying amounts of anisocoria. The 240 photographs were presented in random order to three optometrists for measurement of pupil diameter in each photograph, as well as 118 photographs that were randomly selected to be measured twice by each rater for a total of 358 photographs. Study outcome measures were inter and intra-rater reliability of anisocoria measurements in each photograph, and inter and intra-rater agreement on the presence of anisocoria of one millimetre (mm) or more.

**Results:**

The reliability of anisocoria measurements and agreement on the presence of anisocoria of one mm or more was excellent using the NI-OCT photographs (ICC = 0.93–0.99, k = 0.83–1.0), and decreased considerably using the smartphone photographs (ICC = 0.84–0.90, k = 0.53–0.74), especially for photographs of participants with dark irises (ICC = 0.36–0.82, k = 0 11–0.47).

**Conclusions:**

Using NI-OCT photographs allows for reliable measurement of anisocoria and can aid in the detection of pathological anisocoria, especially in patients with dark irises and children.

## Introduction

Reliable assessment of anisocoria is essential in clinical settings but remains challenging [1–3]. Traditionally, clinicians have relied on manual pupil measurements using a penlight and pupil gauge or pupillary distance (PD) ruler [4, 5], which is very challenging in dim lighting, especially for patients with dark irises, and has demonstrated limited inter-rater reliability [6–14]. Automated infrared pupillometers and eye-tracking devices are considered the gold standard for pupil measurement [15–17]; however, these technologies are rarely available in routine clinical practice.

Smartphone cameras are increasingly used to document anisocoria, but their validity and reliability have not been systematically evaluated. Additionally, photographing the pupils in dim light is very challenging. Near-infrared optical coherence tomography (NI-OCT) is commonly used in ophthalmology clinics for retinal imaging and incorporates near-infrared filters that can visualise pupil diameters. McHugh et al. reported the feasibility of NI-OCT for pupil assessment in a case study using Heidelberg Spectralis OCT during apraclonidine testing [18]. This study compares inter- and intra-rater reliability of anisocoria assessment using smartphones versus NI-OCT imaging.

## Methods

Data collection: In this cross-sectional study, participants were recruited from Hadassah Medical Centre paediatric and adult ophthalmology clinics. After providing informed consent, each participant in the study had their pupils pharmacologically dilated at various time points to create varying amounts of anisocoria using Tropicamide 0.5%.

**Fig. 1:**
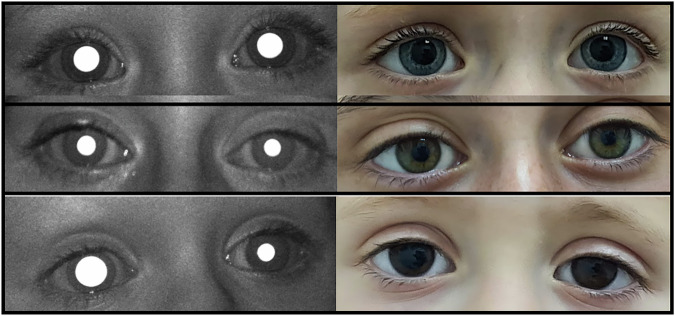
Examples of each eye colour category in NI-OCT and smartphone photographs. Top left. Participant AK (age 3) with blue eyes in NI-OCT photograph. Top right. Participant AK (age 3) with blue eyes in smartphone photograph. Middle left. Participant TK (age 26) with hazel eyes in NI-OCT photograph. Middle right. Participant TK (age 26) with hazel eyes in smartphone photograph. Bottom left. Participant IK (age 5) with brown eyes in NI-OCT photograph. Bottom right. Participant IK (age 5) with brown eyes in smartphone photograph.

Participants provided information on age and sex. Eye colour was categorised at the time of participation into three groups (blue, hazel, and brown). An example of each eye colour from NI-OCT and smartphone cameras is shown in Fig. 1. Participants were excluded from the study if they had irregular pupils, significant corneal opacity, or a large ptosis occluding the pupillary margins.

Simultaneous binocular photographs of each participant were taken at four time points using a Samsung S20 smartphone camera and a Heidelberg Spectralis OCT near-infrared camera at fixed distances determined before the start of the study. Spectralis OCT photos were taken at a distance of 36 cm from the base of the machine lens.

Smartphone pictures were taken using a rear-facing camera on a tripod without flash and with ×5 magnification at a distance of 49 cm.

All photos were taken in the same windowless room under identical lighting conditions, with bright and dim lighting.

Adult participants sat on a chair. Younger children stood on the floor, on a chair, or on the lap of a parent, depending on their height. A video demonstrating the use of the Spectralis OCT to capture images of the pupils of a 14-month-old is provided in the supplementary materials.

The photographs were cropped to show only the eyes of the participants with no other identifying features. Using a random number generator, the photographs were arranged in random order, and half of the photographs were randomly selected to appear twice, non-consecutively.

Three experienced optometrists were asked to manually measure the horizontal pupil diameter (hereafter referred to as pupil size) in each photo using a pupillary distance (PD) ruler, and the anisocoria in the photograph was calculated by subtracting the diameter of the left pupil from the diameter of the right pupil. The raters were blinded and were unaware that some of the photographs appeared twice.

Statistical analysis: All data were exported from Excel to IBM SPSS Statistics 25 and R Studio.

To assess inter-rater reliability, an intraclass correlation coefficient (ICC) analysis was performed based on a mean rating (k = three), absolute agreement, two-way random-effects model, to compare the anisocoria from each photo.

Intra-rater reliability was assessed by comparing the anisocoria measurements of the repeated photographs for each rater. Intraclass correlation coefficient analysis was performed based on a mean rating (k = two), absolute agreement, two-way mixed-effects model, to compare the first and second measurements of anisocoria for each rater in each photo.

To assess the inter- and intra-rater agreement on the presence of anisocoria of one millimetre or more in each photo, the difference in pupil diameter between the eyes was converted to a dichotomous variable that was analysed using Fleiss Kappa and Cohen’s Kappa.

Analyses were repeated within eye colour, sex, and age subgroups for NI-OCT and smartphone photographs. The standard error of measurement (in mm) was calculated for each ICC score and a 99% confidence interval was calculated for each Kappa score.

For clinical applications, relative reliability was deemed excellent, good, moderate, or poor, based on an ICC cut-off score of 0.9, 0.75, 0.5, or <0.5, respectively. The agreement was deemed excellent, substantial, moderate, or poor to fair based on a Kappa cut-off score of 0.8, 0.6, 0.4, or <0.4, respectively.

Sample size calculation: Sample size was calculated using R Studio ‘ICC.sample.size’ package and the function ‘CalculateIccSampleSize’ using an alpha of 0.05, three raters, and a power of 0.8. We calculated a total number of 25 required participants (200 photos, with an additional 100 repeated photos).

Ethical considerations: The study was approved by the institutional Helsinki Review Board (IRB number 0536-22-HMO) prior to data collection. All participants provided written informed consent (or consent of both parents for minors) before participation in the study.

## Results

### Participant demographics

The study involved thirty participants. Eight photos were taken of each participant (four taken with a smartphone and four using the NI-OCT), which resulted in a total of 240 photos. Of these, 118 photos were randomly selected to be assessed twice by each of the raters, making a total of 358 photos included in the study. The photos were assessed by three optometrists from the Hadassah Medical Centre ophthalmology department.

Table 1 provides information on the distribution of the participants based on their eye colour, age, and sex.

**Table 1:** Participant demographics.

Group	Subgroup	*N* (%) [N photos]
**Total**		**30 (100%) [358]**
**Gender**	Male	14 (45.8%) [164]
	female	16 (54.2%) [194]
**Eye colour**	Blue	13 (44.7%) [160]
	Hazel	6 (18.7%) [67]
	Dark brown	11 (36.6%) [131]
**Age**	Children (0–17)	16 (54.5%) [195]
	Mean (sd)	7.74 ( ± 5.71)
	Range	8 months- 16 years
	Adults (18 + )	14 (45.5%) [163]
	Mean (sd)	40.03 ( ± 16.31)
	Range	25–70 years

## Inter-rater reliability results

### Intraclass correlation coefficient (ICC)

The results of the inter-rater reliability analysis comparing the anisocoria measurements of the raters obtained from NI-OCT and smartphone photographs, with subgroup analyses by eye colour, sex, and age group, are presented in Table 2.

**Table 2:** Inter-rater ICC reliability scores for pupil measurements taken from OCT and Samsung S20 photos by eye colour, age group, and sex.

Subgroup	Method	*N*	Pupil size difference (right-left)	
			ICC (99% CI)	SEM (mm)
	**OCT**	**182**	**0.97 (0.96–0.98)**	**0.8**
**Eye colour**	Blue	80	0.95 (0.91–0.97)	0.93
	Hazel	36	0.99 (0.97–0.99)	0.68
	Brown	66	0.98 (0.96–0.99)	0.7
**Age group**	0–17	99	0.97 (0.95–0.98)	0.72
	18+	83	0.97 (0.94–0.98)	0.9
**Sex**	Male	87	0.98 (0.96–0.99)	0.75
	Female	95	0.96 (0.94–0.98)	0.85
	**Samsung S20**	**176**	**0.84 (0.77–0.89)**^**a**^	**1.98**
**Eye colour**	Blue	80	0.94 (0.90–0.97)	1.27
	Hazel	31	0.92 (0.83–0.97)	1.62
	Brown	65	0.62 (0.42–0.78)^a,b^	2.55
**Age group**	0–17	96	0.81 (0.71–0.88)^a^	1.6
	18+	80	0.84 (0.75–0.90)^a^	2.01
**Sex**	Male	77	0.81 (0.68–0.88)^a^	2.4
	Female	99	0.86 (0.81–0.93)^a^	1.58

All *p* values < 0.001.

SEM= standard error of measurement, in millimetres.

^a^Statistically significant difference between the ICC scores of the OCT and smartphone groups, based on the 99% confidence interval.

^b^Statistically significant difference between the ICC between the subgroups of each method, based on the 99% confidence interval.

The ICC scores for the measurements obtained from the NI-OCT photographs show excellent relative reliability between the three raters, with reliability scores of 0.95–0.99 for the measurement of anisocoria. There was no significant change in the reliability scores across the eye colour, age, and sex subgroups.

**Fig. 2:**
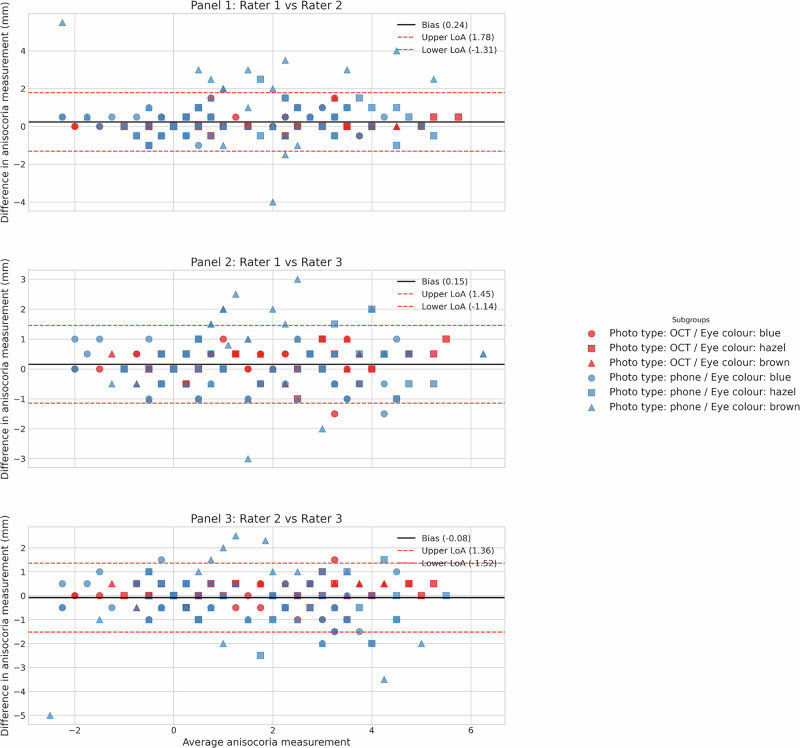
Bland-Altman pairwise comparison of rater agreement of anisocoria measurements by photo type and eye colour. This figure presents a multi-panel Bland-Altman analysis assessing the agreement and interchangeability among three independent raters (Rater 1, Rater 2, and Rater 3) on anisocoria measurements (mm) across all paired observations. For all three comparisons, anisocoria measurements from smartphone photos, particularly of brown-eyed participants show decreased agreement and fall outside the limits of agreement while NI-OCT measurements of participants of all eye colours show only minor systematic disagreement among the raters.

The measurements taken from the smartphone photographs were less reliable than those taken from the NI-OCT photographs but still showed good inter-rater reliability overall (ICC = 0.84). Within the subgroups of the smartphone photographs, inter-rater reliability was excellent for blue- and hazel-coloured eyes (ICC = 0.94 and 0.92, respectively) and decreased significantly for brown eyes (ICC = 0.62). These findings indicate that eye colour has a modifying effect on inter-rater reliability. Comparisons of the raters’ measurements from NI-OCT and smartphone photographs in the blue-, hazel-, and brown-eyed subgroups are shown in Fig. 2.

There was no significant difference in inter-rater reliability between children and adults or between males and females, with the reliability indices remaining similar to the overall inter-rater reliability scores.

### Inter-rater agreement

The results of the raters were classified into a dichotomous variable of a difference in pupil size of ≥1 mm or <1 mm, and agreement was analysed using a Fleiss kappa analysis. The results are presented in the Supplementary Materials in Table A.

The inter-rater agreement on the presence of anisocoria of ≥1 mm from the measurements taken from the NI-OCT photographs is excellent overall and within the eye colour, age, and sex subgroups (κ = 0.93–1.0).

The inter-rater agreement from the measurements taken from the smartphone photographs was moderate overall (κ = 0.53). Agreement decreased significantly from substantial in the blue- and hazel-eyed groups (κ = 0.7) to poor in the brown-eyed group (κ = 0.24). Within the age and sex groups of the smartphone photographs, there is a moderate level of inter-rater agreement (κ = 0.51–0.54) with no significant differences between the groups.

### Intra-rater reliability results

For this analysis, 118 of the photographs were randomly selected to be measured twice by each rater. The raters were unaware that some of the photographs were repeated.

### Intraclass correlation coefficients (ICC)

The intra-rater ICC scores for the three raters are presented in the Supplementary Materials in Table B.

Intra-rater reliability for the measurements taken from NI-OCT photographs was excellent for all three raters, ranging from 0.94 to 0.99. Reliability remained excellent for the NI-OCT photographs in subgroups of eye colour, age, and sex, with no significant differences between the groups.

The intra-rater ICC scores for the smartphone photographs were more varied between subgroups and raters. To assess the impact of sample size on the ICC scores in the brown-eyed smartphone photograph group, the raters were asked to measure the eighteen smartphone photographs of brown-eyed participants a third time (now fifty-four instead of thirty-six measurements), and the intra-rater ICC analysis was repeated. The results of the repeat analysis are shown in Table B (Supplementary Materials) under the original scores for this subgroup. The modifying effect of eye colour is apparent in the reliability scores of the three raters, with good to excellent reliability in the blue-eyed group (0.96) and poor to good intra-rater reliability in the brown-eyed group (0.65, 0.36, and 0.82, respectively, for raters one to three).

There was no significant difference within the smartphone intra-rater ICC scores by age group or sex. The intra-rater reliability indices for these groups ranged from good to excellent for raters one and three (0.85–0.91 and 0.85–0.96, respectively) and moderate to good for rater two (0.69–0.86).

### Intra-rater agreement

The results of Cohen’s kappa analysis are presented in the Supplementary Materials in Table C.

The intra-rater agreement on the presence of anisocoria of ≥1 mm from the measurements taken from the NI-OCT photographs is excellent for each rater in all eye colour, age, and sex subgroups (κ = 0.91–1.0, 0.83–1.0 and 0.88–1.0, respectively).

Within the eye-colour subgroups of the smartphone photographs, the intra-rater kappa scores show excellent agreement in the blue-eyed group (κ = 0.83, 0.84, and 0.84, respectively). In the hazel-eyed group, agreement was excellent for raters one and three (κ = 1.0) and substantial for rater two (κ = 0.72). In the brown-eyed group, intra-rater agreement decreased significantly to moderate for rater one (κ = 0.46) and poor for the other raters (κ = 0.37 and 0.22, respectively). There was no significant difference within the age and sex subgroups of the smartphone photographs, with substantial agreement for all raters (κ = 0.62–0.69).

## Discussion

In this cross-sectional study, inter- and intra-rater reliability were compared for the assessment of anisocoria from photographs taken by the Spectralis NI-OCT and Samsung S20 smartphone. The inter- and intra-rater reliability of the measurement of anisocoria and agreement on the presence of anisocoria of ≥1 mm using NI-OCT was excellent and significantly higher than those from the smartphone photographs.

The inter-rater reliability of anisocoria measurements taken using NI-OCT photographs (ICC = 0.95–0.98, κ = 0.93–1.0) in this study is comparable to reliability indices described for near-infrared pupillometers and eye-tracking devices. Murray et al. found that the inter-device reliability of the RightEye eye-tracking software was 0.99 [15]. Zhao et al. reported the inter-device agreement of the NPi-100 portable pupillometer to be κ = 0.91–0.97 [16], and Stutzman et al. found an agreement of 0.91 between the NPi-200 and NPi-300 [17].

A study by Chopra et al. assessed the inter-rater reliability of a prototype binocular NI-OCT for measurement of pupil diameter, anisocoria, and assessment of a relative afferent pupillary defect (RAPD). The study found excellent reliability for the detection of RAPD with an ICC of >0.98, and an ICC of 0.93–0.97 for measurements of maximum/minimum pupil diameter and anisocoria using the prototype [19]. Our study was able to demonstrate this effect using a standard NI-OCT machine in common use, although RAPD was not addressed.

The imaging technique used in this study was previously described in a case report by McHugh et al. [18] Our study demonstrates the reliability of this method and compares it to assessment with a smartphone. This study also examined the effects of eye colour on pupil assessment reliability and showed that dark irises negatively affect the reliability of anisocoria assessment when not using near-infrared technology [6, 16, 17, 19–21].

These findings have significant clinical implications, as automated pupillometers and eye-tracking technologies are often available in research facilities but not in most clinical settings. Since NI-OCT machines are already in common use in ophthalmology and optometry centres worldwide, they can be used to capture and store photographs of patients’ pupils in addition to routine retinal and optic nerve imaging. These photographs can aid in clinical diagnosis, monitoring, and treatment of patients with various neurological conditions and improve the assessment of anisocoria, particularly in dark-eyed patients, potentially preventing unnecessary pharmacological testing.

This technique can be particularly useful for assessing children, as the photograph is taken from a distance and may feel less threatening. Excellent cooperation was obtained from all children in the study, including an eight-month-old and two-year-old toddlers, and high-quality images were captured using the NI-OCT.

This study has several limitations: (1) The study design only assessed reliability, not the accuracy or validity of the two methods, as no validated pupillometer was available. Thus, assessment of pupils using the NI-OCT has not been validated against the ‘gold standard,’ and sensitivity and specificity of this method for detecting anisocoria could not be determined. (2) NI-OCT was only assessed with the Heidelberg Spectralis NI-OCT and may not be as feasible with other NI-OCT machines. (3) Smartphone imaging was only assessed with the Samsung S20 smartphone. We cannot rule out the possibility that other smartphones may have performed significantly better.

In conclusion, this study highlights the challenges of reliable pupil assessment and detection of anisocoria, particularly in patients with dark irises and young children. It shows that using the Spectralis NI-OCT to capture a binocular image of a patient’s pupils allows for a more reliable assessment than using a smartphone camera. The use of the NI-OCT machine can significantly improve patient care by enhancing the reliability of pupillary assessments with minimal additional effort, as retinal and optic nerve imaging using NI-OCT is routine in most ophthalmology clinics.

Based on the results of the current study, we believe that NI-OCT is a superior alternative to smartphone imaging in the assessment of anisocoria, particularly for patients with dark irises and young children.

## Summary

### What was known before:

- Assessment of anisocoria is extremely challenging in children and patients with dark irises.

### What this study adds:

- Near infrared optical coherence tomography (OCT) is a safe, convenient, and accurate option for assessing anisocoria, particularly for young children.

## Supplementary information

Supplementary tables A-C

Video Demonstrating Technique

## Data Availability

The datasets generated during and/or analysed during the current study are available from the corresponding author on reasonable request.
