# Supplementary material for: Near-infrared OCT imaging for the assessment of anisocoria
Source: Eye (Lond). 2026 May 27;40(11):1728–33. doi: 10.1038/s41433-026-04536-8 (PMC13416134; doi:10.1038/s41433-026-04536-8)
Supplement: Supplementary file 1 — Supplementary tables A-C [file 41433_2026_4536_MOESM1_ESM.docx]

| **TABLE A. Fleiss Kappa agreement scores on the presence of a difference in pupil size ≥1mm from OCT and Samsung S20 photos by eye color, age group, and sex** | | | |  |
| --- | --- | --- | --- | --- |
| **Subgroup** | **Method** | **N** | **Fleiss Kappa score (99% CI)** | |
|  | **OCT** | **182** | **0.93 (0.82-1.0)** | |
| **Eye color** | Blue | 80 | 0.93 (0.75-1.0) | |
|  | Hazel | 36 | 1.0 (0.75-1.0) | |
|  | Brown | 66 | 0.98 (0.77-1.0) | |
| **Age group** | 0-17 | 99 | 0.93 (0.75-1.0) | |
|  | 18+ | 83 | 0.94 (0.75-1.0) | |
| **Sex** | Male | 87 | 0.95 (0.77-1.0) | |
|  | Female | 95 | 0.95 (0.77-1.0) | |
|  | **Samsung S20** | **176** | **0.53 (0.44-0.61)*** | |
| **Eye color** | Blue | 80 | 0.70 (0.53-0.87) | |
|  | Hazel | 31 | 0.70 (0.43-0.96) | |
|  | Brown | 65 | 0.24 (0.06-0.41)*† | |
| **Age group** | 0-17 | 96 | 0.51 (0.42-0.59)* | |
|  | 18+ | 80 | 0.51 (0.40-0.66)* | |
| **Sex** | Male | 77 | 0.54 (0.45-0.67)* | |
|  | Female | 99 | 0.53 (0.43-0.65)* | |
| All p-values ≤0.001  *Statistically significant difference between the ICC scores of the OCT and smartphone groups, based on the 99% confidence interval  † Statistically significant difference between the ICC between the subgroups of each method, based on the 99% confidence interval | | | | |

| **TABLE B. Intra-rater ICC results for pupil measurements taken from OCT and Samsung S20 photos by eye color, age group, and sex** | | | | | | | | | |
| --- | --- | --- | --- | --- | --- | --- | --- | --- | --- |
| **Subgroup** | **Method** | | **N** | **Rater 1** | | **Rater 2** | | **Rater 3** | |
|  |  |  |  | **ICC (99% CI)** | **SEM (mm)** | **ICC (99% CI)** | **SEM (mm)** | **ICC (99% CI)** | **SEM (mm)** |
|  | | **OCT** | **62** | **0.98 (0.95-0.99)**‡ | **0.53** | **0.97 (0.94-0.99)**‡ | **0.58** | **0.99 (0.98-1.0)**‡ | **0.30** |
| **Eye color** | | Blue | 28 | 0.94 (0.84-0.98)‡ | 0.64 | 0.93 (0.80-0.98)‡ | 0.69 | 0.99 (0.97-1.0)‡ | 0.25 |
|  |  | Hazel | 12 | 0.99 (0.95-1.0)‡ | 0.41 | 0.99 (0.96-1.0)‡ | 0.6 | 0.99 (0.97-1.0)‡ | 0.29 |
|  |  | Brown | 22 | 0.98 (0.94-1.0)‡ | 0.47 | 0.98 (0.92-0.99)‡ | 0.55 | 0.99 (0.95-1.0)‡ | 0.37 |
| **Age group** | | 0-17 | 35 | 0.97 (0.91-0.99)‡ | 0.39 | 0.96 (0.87-0.99)‡ | 0.57 | 0.99 (0.97-1.0)‡ | 0.25 |
|  |  | 18+ | 27 | 0.97 (0.91-0.99)‡ | 0.60 | 0.97 (0.91-0.99)‡ | 0.60 | 0.99 (0.97-1.0)‡ | 0.35 |
| **Sex** | | Male | 31 | 0.98 (0.94-0.99)‡ | 0.53 | 0.98 (0.93-0.99)‡ | 0.52 | 0.99 (0.97-1.0)‡ | 0.35 |
|  |  | Female | 31 | 0.96 (0.90-0.99)‡ | 0.55 | 0.95 (0.87-0.98)‡ | 0.62 | 0.99 (0.97-1.0)‡ | 0.27 |
|  | | **Samsung S20** | **56** | **0.90 (0.80-0.95)**‡ | **1.20** | **0.79 (0.60-0.89)**‡* | **1.44** | **0.91 (0.82-0.96)**‡* | **1.10** |
| **Eye color** | | Blue | 28 | 0.96 (0.90-0.99)‡ | 0.71 | 0.96 (0.89-0.99)‡ | 0.70 | 0.96 (0.90-0.99)‡ | 0.77 |
|  |  | Hazel | 7 | 0.90 (0.31-0.99)‡ | 1.26 | 0.69 (-0.21-0.97)€ | 1.77 | 0.95 (0.82-1.0)‡ | 0.80 |
|  |  | Brown | 21 | 0.79 (0.39-0.94)‡ | 1.60 | 0.47 (-0.17-0.82)€*† | 1.80 | 0.77 (0.37-0.93)‡* | 1.50 |
|  |  | ¥Brown | 21 | 0.65 (0.31-0.87)‡*† | 2.67 | 0.36 (-0.03-0.73)‡*† | 2.52 | 0.82 (0.59-0.94)‡* | 2.07 |
| **Age group** | | 0-17 | 32 | 0.91 (0.86-0.98)‡ | 0.8 | 0.86 (0.57-0.94)‡ | 1.33 | 0.96 (0.89-0.99)‡ | 0.74 |
|  |  | 18+ | 24 | 0.85 (0.63-0.97)‡ | 1.40 | 0.71 (0.05-0.88)‡* | 1.64 | 0.90 (0.68-0.97)‡ | 1.15 |
| **Sex** | | Male | 21 | 0.89 (0.62-0.97)‡ | 1.39 | 0.69 (0.48-0.88)‡* | 1.94 | 0.85 (0.52-0.95)‡* | 1.52 |
|  |  | Female | 35 | 0.85 (0.74-0.96)‡ | 1.08 | 0.78 (0.53-0.96)‡ | 0.98 | 0.95 (0.89-0.98)‡ | 0.76 |
| ¥ Repeat ICC analysis of the brown-eyed subgroup with 3 measurements  ‡ P-value< 0.01  € P-value= 0.025  SEM= standard error of measurement, in millimeters  *Statistically significant difference between the ICC scores of the OCT and smartphone groups, based on the 99% confidence interval  † Statistically significant difference between the ICC between the subgroups of each method, based on the 99% confidence interval | | | | | | | | | |

| **TABLE C. Intra-rater Kappa results for detection of a difference in pupil size of more than 1 mm from OCT and Samsung S20 photos by eye color, age group, and sex** | | | | | |
| --- | --- | --- | --- | --- | --- |
| **Subgroup** | **Method** | **N** | **Rater 1**  **Cohen’s Kappa score (99% CI)** | **Rater 2**  **Cohen’s Kappa score**  **(99% CI)** | **Rater 3**  **Cohen’s Kappa score**  **(99% CI)** |
|  | **OCT** | **62** | **0.96 (0.86-1.0)‡** | **0.93 (0.80-1.0)‡** | **0.96 (0.86-1.0)‡** |
| **Eye color** | Blue | 28 | 0.92 (0.71-1.0)‡ | 0.83 (0.54-1.0)‡ | 0.92 (0.71-1.0)‡ |
|  | Hazel | 12 | 1.0 (1.0-1.0)‡ | 1.0 (1.0-1.0)‡ | 1.0 (1.0-1.0)‡ |
|  | Brown | 22 | 1.0 (1.0-1.0)‡ | 1.0 (1.0-1.0)‡ | 1.0 (1.0-1.0)‡ |
| **Age group** | 0-17 | 35 | 0.91 (0.69-1)‡ | 0.83 (0.53-1)‡ | 1.0 (1.0-1.0)‡ |
|  | 18+ | 27 | 1.0 (1.0-1.0)‡ | 1.0 (1.0-1.0)‡ | 0.88 (0.59-1.0)‡ |
| **Sex** | Male | 31 | 0.93 (0.73-1.0)‡ | 0.93 (0.73-1.0)‡ | 1.0 (1.0-1.0)‡ |
|  | Female | 31 | 1.0 (1.0-1.0)‡ | 0.92 (0.72-1.0)‡ | 0.92 (0.73-1.0)‡ |
|  | **Samsung S20** | **56** | **0.74 (0.49-1.0)‡** | **0.64 (0.38-0.90)‡** | **0.64 (0.36-0.92)‡** |
| **Eye color** | Blue | 28 | 0.83 (0.55-1.0)‡ | 0.84 (0.56-1.0)‡ | 0.84 (0.56-1.0)‡ |
|  | Hazel | 7 | 1.0 (1.0-1.0)‡ | 0.72 (0.08-1.0)€ | 1.0 (1.0-1.0)‡ |
|  | Brown | 21 | 0.46 (-0.13-1.0)€ | 0.37 (-0.13-0.87)€* | 0.22 (-0.34-0.78)€* |
|  | ¥Brown | 21 | 0.11 (-0.21-0.44)€*† | 0.17 (-0.15-0.50)€*† | 0.47 (0.15-0.79)‡* |
| **Age group** | 0-17 | 32 | 0.76 (0.42-1.0)‡ | 0.63 (0.24-1.0) | 0.68 (0.34-1.0)‡ |
|  | 18+ | 24 | 0.63 (0.22-1.0) | 0.67 (0.29-1.0) | 0.64 (0.24-1.0)‡ |
| **Sex** | Male | 21 | 0.67 (0.22-1.0)‡ | 0.66 (0.37-1.0)‡ | 0.62 (0.18-1.0)‡ |
|  | Female | 35 | 0.69 (0.42-1.0)‡ | 0.62 (0.18-1.0)‡ | 0.63 (0.31-1.0)‡ |
| ¥ Repeat Fleiss Kappa analysis of the brown-eyed subgroup with three measurements from each rater  ‡ P-value≤ 0.01  € P-value≥0.045  SEM= standard error of measurement, in millimeters  *Statistically significant difference between the ICC scores of the OCT and smartphone groups, based on the 99% confidence interval  † Statistically significant difference between the ICC between the subgroups of each method, based on the 99% confidence interval | | | | | |
